# Perinatal Outcomes of Neonates with Complex and Simple Gastroschisis after Planned Preterm Delivery—A Single-Centre Retrospective Cohort Study

**DOI:** 10.3390/diagnostics13132225

**Published:** 2023-06-30

**Authors:** Renata Jaczyńska, Dariusz Mydlak, Boyana Mikulska, Anna Nimer, Tomasz Maciejewski, Ewa Sawicka

**Affiliations:** 1 Department of Obstetrics and Gyneacology, Institute of Mother and Child, 01-211 Warsaw, Poland; boyana@o2.pl (B.M.); annanimer@hotmail.com (A.N.); tomasz.maciejewski@imid.med.pl (T.M.); 2 Department of Pediatrics Surgery, Institute of Mother and Child, 01-211 Warsaw, Poland; dariusz.mydlak@imid.med.pl (D.M.); ewa.sawicka@imid.med.pl (E.S.)

**Keywords:** gastroschisis, abdominal wall defects, early preterm delivery, caesarean section, prenatal ultrasound, neonate outcome, retrospective cohort study

## Abstract

This research analysed early neonatal outcomes of complex and simple gastroschisis following planned elective preterm delivery in relation to prenatal ultrasound assessment of bowel conditions. A retrospective study of 61 neonates with prenatal gastroschisis diagnosis, birth, and management at a single tertiary centre from 2011 to 2021 showed a 96.72% survival rate with no intrauterine fatalities. Most cases (78.7%) were simple gastroschisis. Neonates with complex gastroschisis had longer hospital stays and time to full enteral feeding compared to those with simple gastroschisis—75.4 versus 35.1 days and 58.1 versus 24.1 days, respectively. A high concordance of 86.90% between the surgeon’s and perinatologist’s bowel condition assessments was achieved. The caesarean delivery protocol demonstrated safety, high survival rate, primary closure, and favourable outcomes compared to other reports. Prenatal ultrasound effectively evaluated bowel conditions and identified complex gastroschisis cases.

## 1. Introduction

Gastroschisis (GS) is a congenital abdominal wall defect located, in most cases, to the right of the insertion of the umbilical cord, in which the intestine—and, rarely, other abdominal organs—are located outside the abdominal cavity with no covering membrane or sac [1]. The incidence of the defect is estimated at nearly 5 per 10,000 live births, and prenatal detection rate exceeds 90% [2,3]. GS is usually an isolated anomaly, it is rarely associated with defects in other organs, and there is no close association with aneuploidies [1,4,5,6]. Pregnancy complicated by gastroschisis is associated with an increased risk of serious perinatal complications: stillbirth, preterm labour, increased length of hospital stay, small-for-gestational-age new-borns (SGA), necrotising enterocolitis (NEC), short bowel syndrome (SBS), neonatal sepsis, and neonatal death (ND) [7].

The origin of gastroschisis has not yet been clearly explained. From a clinical point of view, the pathomechanism of the defect is less important than the presence of accompanying intestinal anomalies (atresia, necrosis, perforation, and volvulus), which qualifies the defect in the cGS (complex gastroschisis) group, as opposed to sGS (simple gastroschisis), where these anomalies are absent [8]. Patients in the cGS group have worse treatment outcomes [2,7]. In addition, progressive damage to the intestine (ischaemia, compression of the mesenteric vessels at the site of abdominal wall defect, and toxic effects of amniotic fluid) results in increased neonatal morbidity and mortality [9,10].

To improve the treatment in children with GS, every stage of the diagnostic and therapeutic process is important—from diagnosis, through proper monitoring, timing and route of delivery, birthplace, timing, and method of defect repair, to postoperative management and long-term care [11,12,13,14,15].

Gastroschisis is a congenital defect in which mortality and morbidity are strongly correlated with intestinal condition at birth [16,17]. The covering of the bowel with fibrous plaque/presence of inflammation (bowel matting) reduces susceptibility to be placed them into the abdominal cavity and the possibility of primary closure, even in the sGS group [18].

The condition of the intestine can be assessed using prenatal ultrasound and can enable proper identification of foetuses/new-borns with a complex type of defect and/or a high probability of postnatal complications, allowing for proper counselling of parents and optimisation of perinatal management.

There is a lot of debate about whether elective preterm delivery (EPD) improves bowel function and whether ultrasound markers can identify a group of new-borns in whom preterm delivery is the optimal management. Research to date has not produced a clear consensus on this issue. The purpose of this study is to analyse the early neonatal outcomes of children with prenatal diagnosis of sGS and cGS in the case of elective early delivery. The secondary objective is the comparative analysis of prenatal ultrasound evaluation of the bowel condition with a surgeon’s assessment and the type of GS (simple vs. complex). 

## 2. Materials and Methods

### 2.1. Study Design

This is a single-centre retrospective cohort study of neonates with a prenatal diagnosis of gastroschisis. The STROBE protocol (dedicated to cohort studies) was used to report the study [19].

### 2.2. Setting

This study included patients diagnosed and managed between 2011 and 2021 at the Department of Obstetrics and Gynaecology and treated at the Department of Paediatric and Adolescent Surgery of the Institute of Mother and Child in Warsaw (Poland).

### 2.3. Participants

The analysis covered all cases of gastroschisis identified during pregnancy, receiving obstetric care at our centre, and giving birth via preterm elective caesarean section. The presence of free-floating bowel loops in the amniotic cavity and the visualisation of a full-thickness right-sided periumbilical abdominal wall defect served as the basis for the diagnosis of the defect. Parents were provided with perinatal counselling by experts in foetal medicine, obstetrics, paediatrics, and paediatric surgery after the defect was identified. All expectant women were made aware that invasive diagnostics were a possibility. An echocardiogram was performed on each foetus.

Of the 61 foetuses, 49 included in the analysis underwent serial examinations performed since the 2nd trimester (long observation, 3 or more ultrasound examinations), and 12 of the 61 foetuses were referred to the clinic and received care 3 to 10 days prior to delivery (short observation, 1–2 ultrasound examinations). After receiving corticosteroid therapy to prevent infant respiratory distress syndrome, all babies were delivered at our facility via caesarean section. The new-borns were then cared for by the departments of surgery, intensive care, and neonatal pathology.

Participants were divided into two groups: simple and complex gastroschisis, based on the work of Molik, who defined cGS as cases accompanied by atresia, necrosis, perforation, and volvulus [8].

### 2.4. Variables

The analysis took into account the following factors: the identification of foetal growth restriction (FGR), delivery mode, birth weight, Apgar score, evaluation of the new-born’s intestine condition, type of primary surgical treatment (primary closure or secondary closure—with implantation of an artificial abdominal wall, i.e., a silo bag), time to repair/closure, intestinal complications (NEC, SBS), and new-born sepsis. Additionally, scores for bowel conditions determined using ultrasound and surgery were contrasted. Time to full enteral feeding (TFEF), length of hospital stay (LOS), intrauterine foetal death (IUFD), and neonatal death were defined as the endpoints (ND).

### 2.5. Data Sources and Measurement

Pregnancy-related medical records, ultrasound images, photographs of the new-borns’ bowel condition, and records of the new-borns’ care were all used. All ultrasound examinations were performed by foetal medicine specialists certified by the Polish Society of Gynaecologists and Obstetricians (PTGiP) and the Foetal Medicine Foundation (FMF, London) using three ultrasound machines: IU22 (Philips), Voluson E8, and Voluson E10 (General Electric). The protocols of the aforementioned associations were followed when conducting US examinations. Ultrasound (US score) and surgical assessment (Surg score) of the bowel condition were carried out according to the following principles: 

#### 2.5.1. Prenatal Ultrasound Assessment of Bowel Condition (Qualitative and Quantitative Assessment)

US 0—no adverse ultrasound signs: normal, stable, and adequate for the gestational age look of EABL (extra-abdominal bowel loops): normal bowel wall (non-hyperechoic, without oedema or/and thickening), free-floating loops without dilatation; no IABL (intra-abdominal bowel loops) dilatation; no gastric dilatation. US 1—any ultrasound-adverse signs or progression: hyperechoic bowel wall or/and oedema or/and thickening; EABL dilatation; lack of lumen of EABL (collapsed bowel), non-free-floating loops with/or without bowel dilatation; IABL dilatation; gastric dilatation.

#### 2.5.2. Postnatal Assessment of Bowel Condition

Surg 0—good: normal bowel without inflammation (no bowel matting), necrosis, atresia, perforation. Surg 1—moderate: slight inflammation or with a visible plaque on the surface (mild bowel matting), always needs to expand (required widening) the abdominal wall defect during primary closure. Surg 2—poor: moderate to massive inflammation with fibrous plaque (severe bowel matting) on the surface and/or necrosis, perforation, atresia, volvulus) stiffness of the intestinal wall EABL.

Hadlock’s formula was used to estimate foetal weight. The FGR group included new-borns with body weight below the third percentile. The diagnosis of FGR did not include measurement of AC (abdominal circumference), which is almost always reduced due to the nature of the defect.

### 2.6. Bias

Three different biases may exist in a retrospective cohort study. The first is selection bias, which can skew the apparent relationship between the exposure and the outcome and results from the way participants are selected or monitored. Information bias, which can result from the subjects being observed, the observers, or the tools being used to assess the results, is the second type. Last but not least, confusion bias can result from other variables that are unrelated to exposure but connected to the outcome. These additional factors have the potential to skew the results of the exposure and create false associations. The likelihood of the aforementioned biases is reduced by the selection criteria used, the use of numerous information sources (including pregnancy records, ultrasound images and descriptions, photo documentation of the new-born, and maternal and child medical records), as well as the inclusion of potentially confounding variables.

### 2.7. Study Population

The cohort of infants with gastroschisis included 66 infants. Three infants who were not monitored at our facility but transferred in utero after 37 weeks of pregnancy and delivered (by caesarean section) at term were not included in the study. The study group also excluded 2 foetuses with vanishing GS (type IV closing GS according to Perrone [20]) because during the observation period, necrosis and severe bowel loop atrophy (EABL) with progressive obstruction had already occurred in the second trimester of pregnancy. These two foetuses were observed under developing gastrointestinal obstruction protocol, not the gastroschisis protocol. 

Sixty-one cases made up the final study group, including 48 infants with sGS and 13 with cGS. The LOS and TFEF were available for 56 children, after excluding 2 deaths and 3 new-borns (2 new-borns due to closing GS (type B and C according to Perrone [20]) with SBS and the need for total parenteral nutrition (TPN), and 1 new-born due to congenital toxoplasmosis for further treatment) transported to another centre—Children’s Memorial Health Institute. 

### 2.8. Statistical Analysis

For continuous variables, data are expressed as means with standard deviation, and categorical variables as number of cases with percent value. The Mann–Whitney U test was applied in order to evaluate the relationship between continuous variables. The Pearson’s Chi-squared test for categorical variables was employed. The agreement between the testing techniques was evaluated using Cohen’s kappa coefficient. The analysis was carried out in the RStudio environment using the R language. *p* values that were less than 0.05 were deemed significant.

## 3. Results

In the examined cohort, all cases of defects were identified during pregnancy. The patients underwent 1 to 12 ultrasound exams while they were expecting. All patients were offered the option of invasive diagnostics after receiving a diagnosis. Eleven (18.3%) foetuses underwent genetic testing, and all the results were normal. Additional malformations were observed in three foetuses: absent septum pellucidum (ASP), arthrogryposis, and cerebellar haemorrhage. 

In our study group, the average gestational age was 34.2 weeks. Elective caesarean sections were used to deliver all the babies. The need for a caesarean section was caused by obstetric complications in two cases (abnormal CTG); in the remaining cases, the indication was the defect itself. Within an hour of birth, almost all infants underwent surgery.

The new-borns were delivered in good condition. Less than 5% of new-borns scored a 6 or 7 on the Apgar scale. Foetal growth restriction was found in 12 (19.67%) new-borns, 11 infants were in the sGS group (22.92%), and 1 infant was classified in the cGS group (7.69%).

Children in the cGS group exhibited a significantly higher prevalence of poor bowel condition than those in the sGS group, as determined by the surgeon. Additionally, they underwent primary closure much less frequently and closing GS was more common (Table 1).

Additionally examined was the type of intestinal complications in cGS. There were 11 cases of atresia (84.6%) in our group, with 9 cases affecting the small intestine and 4 cases affecting the large intestine (two new-borns were diagnosed with both small and large intestine atresia). The cGS group also contained two cases of intestinal necrosis and two cases of perforation. None of the infants were found to have volvulus (Table 2).

Mortality in the study group was just over 3%. A little more than 3% of new-borns also had NEC. Compared to new-borns with sGS (14.53%), new-borns with cGS (69.23%) had a significantly higher rate of sepsis diagnoses. Additionally, the cGS group had noticeably higher LOS and TFEF values. It is interesting to note that for new-borns with complex GS, the median post-conceptual age at discharge was 44.7 weeks, compared to 39.3 weeks for the group of new-borns with simple GS (*p* < 0.001) (Table 3).

The perinatologists described the condition of the intestine in 37 foetuses as a “stable, normal look” (US 0), while in the remaining 24 foetuses they identified any ultrasound signs (US 1). Forty-one new-borns were diagnosed with good intestinal status at birth (Surg 0), seven were classified in the Surg 1 group (moderate), and thirteen were placed in the Surg 2 group (poor). When assessing bowel matting only, this symptom was found in 18 new-borns; however, there was an apparent difference in the frequency of this symptom: in the group of new-borns with sGS, only 16.67% had bowel matting, compared to as much as 76.92% in the cGS group. In the sGS group with evident bowel matting, 7/8 foetuses had ultrasound signs (US 1).The agreement between the prenatal and postnatal bowel assessments was compared in the analysis’ concluding stage. The Surg 1 and Surg 2 groups were combined in this analysis to create a variable signifying poor bowel condition. The surgeon’s assessment and the ultrasound score showed a high degree of agreement at 86.90%. The surgeon’s evaluation of sGS versus cGS had an 82% agreement rate, while in the same case the ultrasound score showed a 78.7% agreement rate (Table 4 and Table 5).

## 4. Discussion

### 4.1. Time and Mode of Delivery

Given the lack of conclusive evidence on the effect of continuous foetal bowel exposure to amniotic fluid and the consequences of preterm delivery, there is currently no consensus on whether a preterm or term birth is more beneficial for a neonate with gastroschisis. Both the optimal delivery time and delivery mode for GS are under discussion.

In our study, as reported by Nitzsche, Mesas Burgos, and Reigstad, all pregnant women included in the analysis delivered prematurely (mean gestational age, 34.2 weeks) via elective caesarean section to prevent intrauterine bowel inflammation and damage caused by vaginal delivery, and to create the best possible conditions for primary closure in the new-born [9,21,22,23].

That approach is predicated on the idea that an advanced gestational age causes intestinal wall inflammation and damage as a result of the influence of inflammatory mediators in the amniotic fluid, which affects neonatal outcome [9,24]. Serra et al. preferred elective caesarean section after 34 weeks of gestation and administering therapy with corticosteroids therapy [10]. Mesas Burgos et al. showed that the optimal age for delivery is between 35 and 36.6 weeks of gestation, as it is associated with a higher rate of primary closure, shorter hospital stay, and reduced need for parenteral nutrition (PN). In addition, elective delivery by caesarean section includes an important aspect regarding the organisation of obstetric–neonatal–surgical care [21].

For some neonatal outcomes, such as feeding, sepsis, and the average number of days spent on mechanical ventilation, a recent systematic review and meta-analysis revealed a trend toward favouring elective preterm delivery. Although they emphasised the need for additional randomised clinical trials (RCTs), the authors did not advocate for elective preterm delivery in gastroschisis [25,26]. In addition, elective preterm delivery appears to be more favourable than spontaneous onset delivery in terms of the incidence of sepsis, short bowel syndrome, prolonged mechanical ventilation, or neonatal death [27]. 

### 4.2. Simple GS and Complex GS

Molik et al. proposed dividing infants born with gastroschisis into a simple and complex type of the defect, as they were able to show that the two groups significantly differed in clinical presentation, postoperative complication rates, increased morbidity, length of hospital stay, and mortality [8]. The prevalence of cGS ranges from 11 to 33, mainly depending on the diagnosis of atresia, which is the most common complication in this group [8,22,28,29,30,31,32,33,34]. It should be noted that the diagnosis of atresia is sometimes possible only in the postoperative period, hence the differences in frequency reported in the literature. In our group, 13 infants were diagnosed with cGS (21.3%) during surgery or in the postoperative period, whereas sGS was diagnosed in 48 infants (78.7%), which is consistent with the literature data [7,30,32,33,34,35].

### 4.3. Foetal Growth Restriction

According to the literature, 17.4–55.5% of foetuses with GS have foetal growth restriction (FGR). Abnormal Doppler parameters in foetuses with gastroschisis are rarely seen, so FGR in this group may be related to protein and fluid loss through the exposed intestine rather than placental insufficiency [29,36,37,38]. In our study group, we observed a smaller number of foetuses with an FGR diagnosis. This might be a result of the planned early delivery. It should be noted that the measurement of abdominal circumference, which is used in weight estimation formulas, is distorted in cases of gastroschisis, making the diagnosis of actual foetal growth restriction difficult, unless it is extreme [39,40].

### 4.4. Primary and Secondary Closure

Each patient with complex gastroschisis is unique. Surgical treatment, including the method of closure and treatment of intestinal damage, must often be tailored to the patient. A universal algorithm for surgical care is not possible due to the variability of the course, “severity” of the lesions, and the complexity of treatment but the following principles are applicable to most patients. Many authors emphasise that primary closure of an abdominal defect, compared to the delayed closure technique, prevents evaporative fluid loss, hypothermia, infection, and inflammation from exposure to the environment and is associated with improved neonatal outcomes: shorter mechanical ventilation time, shorter TPN time, shorter time to start enteral feeding, shorter hospital stay, and lower risk of surgical wound infection compared to the delayed closure technique, lower rate of sepsis. Therefore, primary closure should be favoured when technically possible, but sometimes the distended and matted bowel does not allow for immediate closure [41,42,43,44]. Additionally, if there is any concern about the bowel status, monitoring within a silo is probably safer than immediate reduction and closure. However, silo bags should always be used with caution, especially in cases of complex gastroschisis to avoid complications (bowel wall necrosis due to pressure from the silo ring) and should be placed for the shortest possible time, while carefully monitoring the bowel. That is why some authors advocate that the type of closure should be individualised and taken into account in the procedure that will be necessary to remove the intestinal complications [35]. Ferreira et al. reported in their meta-analysis that primary closure was carried out on every new-born with GS, with no breakdown by defect type in 69% of cases; silo bags were needed in 31% of infants [43]. The likelihood of primary surgery depends on the bowel condition and the presence of concomitant defects, which is why some studies report different primary closure rates in the sGS and cGS groups. However, many works have been published in which the difference is not significant. Primary closure was carried out in 60.1% (7.14–76.47%) and 54.7% (26.56–86.36%) of the cGS and sGS cases, respectively, in a meta-analysis by Bergholz [7]. Primary closure was carried out in 67.4% of all new-borns with early preterm delivery (EPD) in a study by Palatnik—and in a study by Nitzsche, which examined new-borns born prematurely—and was possible on the day of delivery in every case [22,42]. Primary closure was performed on 96.7% of new-borns in our group, all infants with sGS, and 84.62% of infants with cGS; two new-borns were unable to undergo primary closure due to bowel conditions (bowel matting), necessitating the use of a silo bag (secondary closure was performed on the 7th and 9th days of life). Eight neonates with sGS required slight widening of the wall defect to reduce the bowel into the abdominal cavity due to moderate bowel matting. In the cGS group, only three neonates did not need the extension of the wall defect. Sawicka et al. analysis of neonates with GS between 2000 and 2010 showed that the only factor affecting prognosis was the time from birth to surgery (<3 h) [15]. In our study, abdominal wall closure was performed within the first hour of life in 57 new-borns, within 2 h in 1 new-born, and within the first 5 h of life in 1 baby.

### 4.5. Intestinal Complications in cGS

Intestinal atresia, which occurs between 23.8% and 94.7% of the time in complex gastroschisis, is the most frequent intestinal complication [8,30,34,35,45,46]. Jejunal atresia accounts for about 80% of atresia cases. cGS new-borns frequently have multiple coexisting complications [28,47]. Bowel atresia is identified in three scenarios [35]. The first and most common is when atresia is diagnosed at the time of first assessment of the bowel. The second is a suspected atresia or stenosis that cannot be confirmed due to severe bowel matting but is confirmed later. The third scenario is a stenosis or atresia diagnosed in a patient initially classified as simple gastroschisis. In our group, six new-borns were diagnosed with atresia at the time of the first intestinal evaluation, two according to the second scenario, and three in the postoperative period, originally classified as simple GS. This is consistent with the findings of other authors, who claim that atresia can be overlooked during the initial surgery in roughly 123–40% of instances because it is challenging to accurately assess each individual intestinal loop due to bowel matting [30,35,45,47,48]. According to studies, the incidence of intestinal perforation in cGS ranges from 2.9% to 30% [28,29,34,49,50], and the incidence of necrosis is between 14.2% and 43.3% [34,49,50]. In our group, two (15.4%) new-borns had perforation and necrosis diagnoses. Our results are identical to Lapp’s report [30]. We did not find volvulus in our group. In the literature, the incidence of this complication ranges from 0.97% to 37.5% [31,34,49].

In the preoperative period, three neonates in the cGS group were diagnosed with closing gastroschisis (hypoplastic/shrinkage of the extra-abdominal bowel and small defect size) [46]. 

Two of the neonates had bowels with massive inflammation and fibrous plaque (peel formation), which made primary closure impossible.

Bowel resection is necessary in the great majority of patients with complex gastroschisis [35]. In our group of new-borns with cGS, intestinal resection was performed in five neonates (38.46%) during the initial surgery. All three neonates with closing gastroschisis required small bowel resection. Two of them had significant bowel resection resulting in short bowel syndrome. 

In deciding on primary anastomosis, three conditions must be met: absence of anastomotic tension, good blood supply, and absence of distal obstruction. Then, the atresia may be corrected immediately after birth at the time of abdominal primary closure or silo placement [35]. In our cGS group, primary anastomosis was performed on six new-borns, and seven underwent staged treatment with ileostomy/colostomy. One new-born required improvement of small bowel anastomosis.

Post-closure reoperation was performed in four neonates—three of them with atresia diagnosed postoperatively (initially classified as a sGS) and one of them due to adhesion-related small bowel obstruction.

### 4.6. NEC

According to recent studies, NEC affects 4–5% of children with gastroschisis. Only one-quarter of these infants need surgery for NEC, and the course of this complication is typically mild in most patients. [51]

Some authors point out that most episodes of necrotising enterocolitis are seen in patients with simple rather than complex gastroschisis [18,51]. In his meta-analysis, Bergholz showed the presence of NEC in 14% of new-borns with sGS and 8% of those with cGS [7]. When analysing the results of studies involving groups of preterm new-borns, in a study published by Palatnik et al., in the EPD (early preterm delivery) cohort (31.0–34.6 weeks), NEC was reported in 4.6% of the entire group of new-borns with GS [42]. In a study by Dekonenko et al., who also analysed the EPD group but by defect type, NEC occurred in 6 and 14% of sGS and cGS cases, respectively [48].

In our group, NEC occurred in two new-borns, which was a lower frequency in both sGS and cGS groups compared to the results of most authors.

### 4.7. Short Bowel Syndrome

The presence of intestinal complications makes short bowel syndrome significantly more likely to occur in people with complex GS (SBS). According to the meta-analysis data, the prevalence of SBS varies depending on the defect type, averaging 27.0% (7.1–58.5%) for cGS and 1.68% (0–2.97%) for sGS [7,8]. The impact of short bowel syndrome on the overall health and quality of life of children is significant, and the long-term effects of SBS are very serious, with child mortality rates ranging from 27.5% to 37.5% [52]. Therefore, it is necessary to pay attention to signs when performing prenatal ultrasound that can predict the occurrence of this complication. It is worth considering early delivery to avoid or mitigate the health consequences of having to perform a major bowel resection [7]. The particular form of the defect, closing GS, is a risk factor for SBS even in the sGS group. In our analysis, SBS occurred in two new-borns only in the cGS group as a result of closing of the abdominal wall defect (closing GS). There was no need for bowel resection leading to SBS in another two new-borns who were also diagnosed with prenatally suspected closing GS, confirmed after birth. Palatnik presented a similar frequency in a comparable age range. In her research, 4.6% of infants with GS who were born prematurely experienced SBS, and Shamshirsaz found SBS in 1 of every 10 study-involved new-borns in the early delivery group [42,53].

### 4.8. Sepsis

The EPD group has a wide range of sepsis incidence, from 6.8 to 40% [42,53]. Sepsis frequency was presented by Molik et al., with values for sGS and cGS of 21.1% and 46.9%, respectively [8]. Similar rates of sepsis were reported in the meta-analysis by Bergholz: 51% (20–68.75%) for cGS and 20.7% (8.57–33.33%) for sGS [7]. In Girsen’s study, sepsis affected 33.9% of infants born before 37 weeks of gestation and 23.9% of those born after 37 weeks. A difference was observed in the incidence of sepsis between the subgroup of infants with planned early delivery and in those whose preterm labour began spontaneously—28 and 40%, respectively. Although the cGS group had a higher incidence of sepsis in our study, incidence of sepsis in the entire group was either similar or lower compared to data published by other authors [27].

Elective preterm delivery by caesarean section, according to Goldstein et al. and Landish et al., appeared to reduce the incidence of sepsis, and there was a trend toward reducing the number of days on mechanical ventilation without increasing LOS or mortality [25,26]. However, Shamshirsaz et al., conducting an RCT, decided to stop the study due to the occurrence of sepsis in 4/10 neonates in the EPD group [53]. It should be noted that, based on published data from other authors, a 40% incidence of sepsis does not appear to be a poor result. However, the aforementioned study does not specify whether the cases of sepsis occurred in the group of new-borns with sGS or cGS.

### 4.9. LOS

Since there is a noticeable difference in LOS between the two GS types, authors frequently present LOS results according to gestational age at delivery and the type of defect (sGS vs cGS). The mean LOS in the studies cited in Bergholz’s meta-analysis was 37.83 days (range: 23–43.7 days) for sGS and 116.96 days (range: 90.4–138.25) for cGS, respectively [7]. Our entire group’s LOS values were 42.3 ± 26.5, which are similar to those reported by Palatnik—45.9 ± 34.8 days—for the “early preterm delivery” group (without splitting into sGS and cGS), and were shorter than those in Shamshirsaz’s study, which reported an average LOS of 70.5 days in a group of new-borns born at a similar gestational age [42,53]. Comparing the LOS in the group of children with sGS, it was similar to the data reported by Dekonenko, Molik, and Kuleva. However, a shorter hospital stay achieved in the cGS group [8,48,49].

### 4.10. TFEF

Preterm delivery may offer benefits related to a shorter time on total parenteral nutrition (TPN), according to a meta-analysis by Landisch et al. [25]. Compared to data on preterm new-borns examined by Palatnik et al., the mean TFEF for the entire study group in our study was shorter [42]. The mean TFEF for sGS in the meta-analysis by Ferreira and Bergholz was 26–27 days, which was comparable to the findings of our analysis. The average time in the cGS group, however, ranged from 116 to 165 days [7,43]. Our result in this group was significantly lower (58.1 days) and similar to Molik’s study, in which full enteral nutrition was achieved after 50 days (ranging from 21 to 113 days) [8].

### 4.11. Neonatal Death and IUFD

The overall mortality rate in the entire group of children with gastroschisis ranges from 3 to 8.71% [30,34,54]. Mortality in the sGS group ranges from 0 to 3.4%, and from 8.7 to 28% in the cGS group [7,8,28,30,55]. In studies analysing deaths of children born prematurely, mortality was estimated without dividing into sGS and cGS groups, but according to gestational age at delivery: mortality rate was assessed as 6.8%, 5%, and 5% in the early preterm, late preterm, and term delivery cohorts, respectively [42]. While mortality rates for new-borns with sGS in our study group were comparable to those reported in the literature, they were lower (7.69%) in the group with cGS.

South et al.’s meta-analysis reported a 4.48% incidence of IUFD in gastroschisis [56]. In the most recent meta-analysis, Ferreira et al. presented more favourable results (0.34%) [43]. No IUFD was noted in our data, most likely as a result of the early delivery and the careful and precise foetal monitoring protocol used. IUFD was also not reported in studies on new-borns with EPD [22,42,53]. By employing a protocol based on repeated ultrasound examinations, CTGs, and education of the pregnant woman regarding potential complications and the significance of tracking foetal motor activity, Pery et al. were able to lower the IUDF rate from 5.3 to 2.2% [57].

### 4.12. Bowel Matting as the Only Criterion for Evaluation of Bowel Condition in sGS

The gastroschisis prognostic score (GPS), developed by Cowan et al., is one of the few GS severity classification systems. It is based on examining and correlating bowel appearance at birth with clinical outcomes and has been prospectively validated as a prognostic indicator of mortality and morbidity for the entire spectrum of gastroschisis [16].

Three of the four components of this score, atresia, necrosis, and perforation, are not applicable to patients with sGS. The only marker of a new-born’s bowel condition in the sGS group that can be used is bowel matting. Severe matting was found to be an independent predictor of morbidity when evaluating the usefulness of the GPS score [16]. Our study showed a significant difference in the occurrence of this sign between sGS and cGS groups. 

According to other authors, bowel matting can be a symptom suggestive of cGS, but it is not an independent risk factor negatively affecting prognosis; however, neonates diagnosed with severe matting had a higher risk of failure of primary closure attempts [18,48]. Both neonates in the cGS group in our analysis who failed primary closure were diagnosed with severe bowel matting. 

### 4.13. Diagnosis Agreement

We have no influence on the incidence of cGS, but prenatal diagnosis and monitoring of the defect allows for the identification of a group of new-borns at high risk of severe complications even before birth. Because of the significant differences in morbidity and mortality in the cGS group compared to sGS, research has focused on using ultrasound features that could be potential signs of cGS. 

The task of the perinatologists is to identify foetuses with a suspected complex type of GS, properly counsel parents, and ensure optimal perinatal management in collaboration with surgeons. 

At the same time, for the surgeon, the most important factor in determining the possibility of primary abdominal wall closure is the “state of pathology of the bowel”, which is displaced outside the abdominal cavity, and the coexistence of other bowel defects [16,17]. Therefore, we conducted an analysis of the agreement of diagnoses between ultrasound evaluation, surgeon’s postnatal evaluation, and GS type. The obtained agreement rate of about 80% allows us to conclude that a proper diagnostic process was carried out, and ultrasound evaluation is an appropriate tool for identifying foetuses with an increased risk of intestinal complications. 

Planned early delivery at the time when the unfavourable ultrasound signs appear is a compromise between foetal maturity and the bowel condition and provides an opportunity for optimal treatment in a given situation. If the inflammatory bowel damage observed in patients with gastroschisis is progressive, this would suggest that preterm delivery and treatment of the new-born immediately after birth could interrupt this process and improve both short- and long-term outcomes in these children. The key, however, is to avoid comorbidity caused by early delivery, which makes timing very important [21,30].

At our centre, over the years and based on our own experience, we developed a management regimen that is based on delivery at the time when the condition of the bowel, assessed using close ultrasound monitoring, begins to change (to deteriorate), in order to prevent progression that would make primary closure impossible. Planned delivery at the right time can also prevent severe forms of the defect—closing/vanishing gastroschisis—and reduce the risk of short bowel syndrome. 

The obtained outcomes may be the result of the developed protocol for prenatal monitoring, premature delivery, and postnatal management of the new-born. 

Based on these, it can be assumed that despite elective preterm birth, most of the neonatal outcomes analysed in our study are similar to or better than those reported in analyses of groups of preterm and term-born babies. 

Despite the ongoing controversy regarding obstetric management of pregnancies with gastroschisis, especially the choice of time and route of delivery, our analysis suggests that preterm, elective caesarean section is the optimal management strategy.

## 5. Limitations

This study’s primary drawback is that it is retrospective in nature. As a result, it was impossible to determine how the defect would develop naturally or how the observed changes in bowel condition would change if the pregnancy was continued. Additionally, we did not have a comparison group with expectant management/term delivery, so we compared the results with the literature data. 

A prospective study and observation of the changes taking place within the eviscerated bowel could allow for gaining knowledge on the occurrence of successive changes and their impact on the postnatal condition of the bowel, type of abdominal wall closure, LOS, and TFEF. It would help to choose the timing of delivery of pregnancy so as to achieve the longest possible gestation period but at the same time prevent intestinal damage that would make single-stage treatment impossible, leading to worse neonatal outcomes.

However, the idea of randomised controlled trials as the sole source of evidence is not practical due to the variability of the course, the “severity” of the lesions, and the complexity of treatment. It seems more valuable to implement guidelines for clinical practice based on the experience of multidisciplinary expert groups and to conduct multicentre observational studies. 

This study has a number of advantages. Data were gathered at a single tertiary facility where neonatal care was standard throughout the study period and foetal monitoring adhered to a predetermined protocol. The results of neonates born prematurely by elective caesarean section, divided into the sGS and cGS groups, are also important information provided by our study.

## 6. Conclusions

In comparison to the cited studies examining cases of neonates born at term, as well as in the EPD scheme, we achieved better results in terms of primary closure rates and incidence of NEC, SBS, IUFD, ND, and sepsis in the sGS group. In the cGS group, we demonstrated shorter LOS and TFEF, whereas the sGS group’s results were comparable to those of the cited papers. We only obtained worse results in terms of the occurrence of sepsis in the cGS group. In our study, we presented a high agreement rate between the perinatologist’s ultrasound assessment, the surgeon’s clinical assessment, and the final classification of the defect into the sGS or cGS group. This leads us to the conclusion that prenatal ultrasound examination enables us to evaluate the condition of the intestine and identify foetuses with suspected complex gastroschisis.

## Figures and Tables

**Table 1:** Comparison of variables between the sGS and cGS groups.

Variable		Simple Gastroschisis *N* = 48		Complex Gastroschisis *N* = 13		All *N* = 61		*p*
		*n*/M	%/SD	*n*/M	%/SD	*n*/M	%/SD	
Mother age		25.4	5.08	24.1	5.16	25.1	5.08	0.372
Gestational age (weeks)		34.3	0.811	33.9	0.94	34.2	0.848	0.134
Neonatal birth weight		2131	350	1998	349	2102	351	0.238
Foetal growth restriction		11	22.92%	1	7.69%	12	19.67%	0.406
Apgar score	6	0	0.00%	1	7.69%	1	1.64%	0.352
	7	2	4.17%	0	0.00%	2	3.28%	
	8	3	6.25%	1	7.69%	4	6.56%	
	9	10	20.83%	2	15.38%	12	19.67%	
	10	33	68.75%	9	69.23%	42	68.85%	
Time to repair (hours)	1	47	97.92%	12	92.31%	59	96.72%	0.136
	2	0	0.00%	1	7.69%	1	1.64%	
	5	1	2.08%	0	0.00%	1	1.64%	
New-born bowel condition	Good	39	81.25%	2	15.38%	41	67.21%	<0.001
	Moderate	6	12.50%	1	7.69%	7	11.48%	
	Poor	3	6.25%	10	76.92%	13	21.31%	
Bowel matting	No	40	83.33%	3	23.08%	43	70.49%	<0.001
	Mild	5	10.42%	2	15.38%	7	11.48%	
	Severe	3	6.25%	8	61.54%	11	18.03%	
Closing gastroschisis	Yes	1	2.08%	3	23.08%	4	6.56%	0.037
	No	47	97.92%	10	76.92%	57	93.44%	
Primary closure	Yes	48	100.00%	11	84.62%	59	96.72%	0.059
	No	0	0.00%	2	15.38%	2	3.28%	
Need to widen the wall defect before closure		8	16.67%	10	76.92%	18	29.50	<0.001
Short bowel syndrome		0	0	2	15.38%	2	3.28%	N/A
Ileostomy/colostomy		0	0	7	53.85%	7	11.48%	N/A
Post-closure reoperation		0	0	4	30.77%	4	6.56%	N/A
Bowel resection		0	0	5	38.46%	5	8.20%	N/A

**Table 2:** Intestinal complications in cGS.

Bowel Complication	*n*	%
Atresia	11	84.62
Necrosis	2	15.38
Perforation	2	15.38
Volvulus	0	-

**Table 3:** Comparison of results (outcome) between the sGS and cGS groups.

Outcome Variable	Simple Gastroschisis *N* = 48 (21.3%)		Complex Gastroschisis *N* = 13 (78.7%)		All *N* = 61		*p*
	*n*/M	%/SD	*n*/M	%/SD	*n*/M	%/SD	
Neonatal death	1	2.08%	1	7.69%	2	3.28%	0.897
NEC	1	2.08%	1	7.69%	2	3.28%	0.897
Sepsis	7	14.58%	9	69.23%	16	26.23%	<0.001
Transfer	1	2.08%	2	15.38%	3	4.92%	-
Time to full enteral feeding	24.1	11.7	58.1	29.3	30.2	20.6	<0.001
Length of hospital stay	35.1	16.9	75.4	37.3	42.3	26.5	<0.001
Post-conceptual age of discharge from the hospital (weeks)	39.3	2.42	44.7	4.88	40.3	3.6	0.001

**Table 4:** Comparison of perinatologist’s and surgeon’s assessments of the intestinal condition according to the presence of sGS and cGS.

	Bowel Condition	Simple Gastroschisis *N* = 48	Complex Gastroschisis *N* = 13	*p*	Cramér’s V
Perinatologist’s assessment (foetus)	US 0	36 (75.00%)	1 (7.69%)	<0.001	0.59721
	US 1	12 (25.00%)	12 (92.31%)		
Surgeon’s assessment (new-born)	Surg 0	39 (81.25%)	2 (15.38%)	<0.001	0.60645
	Surg 1 or Surg 2	9 (18.75%)	11 (84.62%)		

**Table 5:** Diagnosis agreement rates.

	Agreement	Kappa
Prenatal intestinal condition (perinatologist) vs. complex/simplex	78.70%	0.514
Neonatal intestinal condition (surgeon) vs. complex/simplex	82.00%	0.551
Neonatal intestinal condition (surgeon) vs. prenatal intestinal condition (perinatologist)	86.90%	0.717

## Data Availability

Data are available upon request to the corresponding author.
